# Additional Common Polymorphisms in the *PON* Gene Cluster Predict PON1 Activity but Not Vascular Disease

**DOI:** 10.1155/2012/476316

**Published:** 2012-05-22

**Authors:** Daniel S. Kim, Amber A. Burt, Jane E. Ranchalis, Rebecca J. Richter, Julieann K. Marshall, Jason F. Eintracht, Elisabeth A. Rosenthal, Clement E. Furlong, Gail P. Jarvik

**Affiliations:** ^1^Department of Genome Sciences, University of Washington School of Medicine, Seattle, WA 98195, USA; ^2^Department of Medicine, Division of Medical Genetics, University of Washington School of Medicine, P.O. Box 357720, Seattle, WA 98195, USA; ^3^Department of General Medicine, Virginia Mason Medical Center, Seattle, WA 98101, USA

## Abstract

*Background*. Paraoxonase 1 (PON1) enzymatic activity has been consistently predictive of cardiovascular disease, while the genotypes at the four functional polymorphisms at *PON1* have not. The goal of this study was to identify additional variation at the *PON* gene cluster that improved prediction of PON1 activity and determine if these variants predict carotid artery disease (CAAD). *Methods*. We considered 1,328 males in a CAAD cohort. 51 tagging single-nucleotide polymorphisms (tag SNPs) across the *PON* cluster were evaluated to determine their effects on PON1 activity and CAAD status. *Results*. Six SNPs (four in *PON1* and one each in *PON2/3*) predicted PON1 arylesterase (AREase) activity, in addition to the four previously known functional SNPs. In total, the 10 SNPs explained 30.1% of AREase activity, 5% of which was attributable to the six identified predictive SNPs. We replicate rs854567 prediction of 2.3% of AREase variance, the effects of rs3917510, and a *PON3* haplotype that includes rs2375005. While AREase activity strongly predicted CAAD, none of the 10 SNPs predicting AREase predicted CAAD. *Conclusions*. This study identifies new genetic variants that predict additional PON1 AREase activity. Identification of SNPs associated with PON1 activity is required when evaluating the many phenotypes associated with genetic variation near PON1.

## 1. Introduction

Paraoxonase 1 (PON1) is a liver-produced glycoprotein enzyme bound to the surface of high-density lipoprotein (HDL) whose activity is consistently correlated with atherosclerotic vascular disease and end-organ damage [1–3]. PON1 is at least partially responsible for the inhibitory effects of HDL on low-density lipoprotein (LDL) peroxidation [4–6] and also has been demonstrated to hydrolyze oxidized lipid or lipid hydroperoxides in LDL [7]. Accordingly, Watson et al. reported that inactivation of PON1 reduced the ability of HDL to inhibit both the oxidation of LDL and the interaction between macrophages and endothelium [6], both likely key factors in the inflammatory changes underlying atherogenesis. It has also been shown that PON1-deficient mice cannot neutralize the oxidized LDL lipids and have an increased susceptibility to organophosphate toxicity and coronary heart disease (CHD) [8, 9]. Finally, PON1 activity appears to play a role in maintaining the endothelial-atheroprotective effects of HDL [10].

There are four currently established functional common *PON1* single-nucleotide polymorphisms (SNPs) amongst the nearly 200 SNPs in the gene [11]: two missense mutations (*PON1_Q192R _*[rs662] and *PON1_M55L _*[rs854560]) and two that alter promoter activity (*PON1_-108C/T _*[rs705379] and *PON1_-162A/G _*[rs705381]). *PON1_-108C/T_* has the largest effect, altering expression likely due to modification of an Sp1 binding site [12]. Rare functional variants have also been identified [13].

While PON1 activity is predictive of vascular disease, studies investigating the role of *PON1* SNPs in vascular disease have been contradictory [14–18]. A recent meta-analysis of 88 case-control studies by Wang et al. found that *PON1_Q192_* was correlated with CHD [19]. However, removal of smaller studies from the meta-analysis resulted in none of the functional *PON1* SNPs having significant association with CHD, thereby replicating the results of past meta-analyses [20–22]. Similarly, our own past investigations have found that while PON1 enzyme levels are predictive of carotid artery disease (CAAD), the genotypes at the four common functional SNPs fail to predict CAAD status [2, 3]. However, studies of CAAD or ischemic strokes are generally more positive for associations with the *PON1* functional SNPs [18, 23–26] than those for CHD. It should be noted that these studies generally have small sample size and several of such studies reported negative results [27, 28].

PON1 has broad substrate specificity and is protective against exposure to toxic organophosphorus insecticides [29]. For biological purposes, PON1 activity is generally measured with regard to the rate of hydrolysis of paraoxon, diazoxon, and phenylacetate (arylesterase activity) [30, 31]. These are termed POase, DZOase, and AREase activities, respectively. AREase enzymatic activity is unaffected by the functional *PON1_Q192R_* polymorphism, thus making it the best reflection of the levels of PON1 protein [32].

PON1 activity has also been linked to a number of other health-related phenotypes in addition to vascular disease and diabetes [33]. For example, PON1 also influences the metabolism of a variety of drugs, including statins, in addition to its aforementioned properties of reducing oxidized LDL and breaking down pesticides [34]. *PON*1_Q192R_ is a reported determinant of clopidogrel efficacy [35], although this result has not been replicated [36] and remains controversial. PON1 has also been associated with diverse diseases [37]. The *PON1_L55M_* polymorphism has been repeatedly associated with Parkinson's disease [38–40], including a meta-analysis [41], but null results have also been reported [42]. Recent meta-analyses reported the association of *PON1* coding SNPs and breast cancer [43, 44]. Both PON1 activity and genotypes have been associated with age-related macular degeneration [45–51]. PON1 activity is reportedly lower in subjects with systemic lupus erythematosus (SLE) [48, 52–54]. Finally, diabetes is associated with both reduced PON1 activity and *PON1* genotypes [55].

*PON1* is one of three paraoxonase gene family members, located in a gene cluster on chromosome 7q21.3-22.1. All of the paraoxonases have antioxidant activity [56]. *PON1* and *PON3* share similar functions in association with HDL as described previously; however, *PON3 *has lower expression levels [57]. In contrast, *PON2* is ubiquitously expressed in human cells [58], particularly in endothelial and aortic smooth muscle cells [59]. *PON2* polymorphisms have also been associated with CHD [58, 60]. In addition, all three *PON* gene products have been reported to hydrolyze the quorum sensing factor of *Pseudomonas aeruginosa N*-3-oxododecanoyl homoserine lactone (3OC12-HSL)[61], with PON1 and 2 enzymes specifically being shown in animal knock-out studies to be protective against *p. aeruginosa* infection [62, 63].

Carlson et al. previously performed a tagSNP analysis of the *PON1, 2, *and *3* gene cluster for association with AREase activity and CAAD status in an overlapping, but much smaller, cohort (*n* = 500 versus 1328) [27]. That study found evidence that additional functional SNPs likely exist in *PON1*, but that the majority of the genetic effect on AREase variation was explained by the four functional SNPs previously described. They did not find evidence for *PON2 *or *PON3* SNPs predicting additional AREase activity.

However, the investigation by Carlson et al. still left a large portion of the variation in PON1 activity unexplained. Thus, the goals of this study are to followup on these previous results and utilize an enlarged cohort and denser tagSNP genotyping to attempt to identify novel common SNPs in the *PON* gene cluster that associate with PON1 activity and/or predict CAAD.

## 2. Methods

### 2.1. Sample

The study population for this analysis consisted of 1,328 samples from the previously described Carotid Lesion Epidemiology And Risk (CLEAR) study [2, 3, 64]. Only Caucasian males were analyzed due to underrepresentation of female and minority samples in this primarily Seattle-Veterans-based cohort. Current smoking status and reported ancestry were obtained by self-report. Ancestry was confirmed using STRUCTURE with three ancestral groups [65]. CAAD status was determined via ultrasound of the internal carotid arteries, with cases defined as having >50% stenosis in either artery or a relevant procedure on their carotid arteries in their medical history. Controls had <15% stenosis in both arteries. 88 subjects had intermediate stenosis (15–49%) and were not included for prediction of CAAD, though they were included for prediction of PON1 enzyme activity.

### 2.2. Genotyping and PON1 Phenotypes

The four known functional *PON1* SNPs, *PON1_Q192R_*, *PON1_M55L_*, *PON1_−108C/T_*, and *PON1_−162A/G_* and two SNPs identified as potentially predictive by Carlson et al. but not represented on the CVD chip, *PON1_−909_* (rs854572) and rs3917510 [27], were genotyped using previously described methods [12, 66]. An additional 86 SNPs in *PON1*, *PON2,* and *PON3* cluster were genotyped using the Illumina HumanCVD BeadChip (http://www.illumina.com/products/humancvd_whole_genome_genotyping_kits.ilmn). Duplicate genotyping for 34 individuals showed 99.7% consistency in calls. The *PON* cluster genotypes were filtered with a minor allele frequency cutoff of 1% and did not show deviation from Hardy-Weinberg equilibrium at the *P* < 10^−4^ level. Rs3917564 was also found to be predictive by Carlson et al. and was genotyped by the CVD chip but was not included in the full analysis due to low minor allele frequency (C/T, C allele frequency = 0.008).

The PON1 POase, DZOase, and AREase activities were measured by a continuous spectrophotometric assay with lithium heparin plasma, as previously described [66]. AREase activity was measured in duplicate and averaged. AREase was utilized as the primary measured outcome of *PON* gene cluster variation, due to its closer correlation with protein levels. POase activity is largely determined by the PON1_Q192R_ missense polymorphism, which predicts over 70% of its variance [2].

### 2.3. Analysis

LDselect was used to create tagSNPs from the 86 *PON1*, *PON2,* and *PON3* SNPs genotyped on the Illumina HumanCVD chip [67]. Functional annotation for these SNPs were taken from SNP-Nexus [68]. 51 bins were created, using a linkage disequilibrium (LD) *r* ^2^ threshold of 0.64. The first 13 of these bins, corresponding to the *PON1 *gene, had multiple SNPs within them, while the remaining bins consisted of singletons. One SNP from each bin was randomly included in the regression analysis for a total of 51 SNPs in the *PON *gene cluster. These 51 SNPs did not include the four functional SNPs, which were included in the analysis separately.

We also made an effort to independently replicate SNPs identified as predictive of PON1 activity by Carlson et al. [27]. As our full sample overlaps with that smaller sample, these were tested in a nonoverlapping sample of 523 subjects with complete genotype and phenotype data which were not available at the time of that study.

Regression analysis was done in R (http://www.r-project.org/) using the standard regression tools available. Genotypes were coded using an additive model. Stepwise linear regression was performed, and model comparison was done using Akaike's Information Criterion (AIC) to examine the fit of each model, beginning with a base model that included current smoking status, age, and the genotypes for the four functional PON1 SNPs as covariates [2, 3, 27]. SNPs that are included in the final model increased the ability of the model to predict the dependent variable. Statin drug use can influence *PON1* expression, and this appears to be influenced by *PON1_−108_* genotype [69]. However, statin drug use could not be included as a covariate due to confounding with CAAD status; the preferential use of statins in cases can lead to an erroneous estimation of statin effects on PON1 activity.

## 3. Results

The sample included 1,328 males with a mean age of 67.8 years; 16.5% of participants reported being current smokers. The subjects included 596 cases and 644 controls considered in the prediction of case status as well as 88 subjects with carotid stenosis between 15–49% who were considered only for the genotype effects on PON1 activity. Cases had a mean censored (CAAD onset) age of 66.5 years and mean current age at enrollment age of 70.9; controls had a mean current age of 64.6 years. The rates of current smoking and statin use, respectively, were 25.8% and 64.7% for cases and 9.6% and 19.5% for controls. Descriptions of the 51 tag SNPs for the *PON* gene cluster are available in Table 1. The AREase activity showed an approximately normal distribution, with a mean of 134 U/I and standard deviation of 51.8.

A regression model containing functional *PON1* SNPs (*PON1_Q192R_, PON1_M55L_, PON1_-108C/T_, *and* PON1_-162A/G_*), age, and current smoking status explained 25.2% of the variance in AREase activity. To explore the possibility of novel SNPs influencing AREase activity, we examined a best-fit model utilizing the stepwise regression including the aforementioned variables plus the 51 tagSNPs. AIC was used to assess whether the additional SNP provided a better fit to the prediction of AREase activity. Only SNPs that added to the predictive power of the best-fit model were kept; others that did not influence the model were discarded. In addition to the four functional SNPs, age, and current smoking, six SNPs were retained in the best-fit model. Together with the original 4 functional SNPs, these additional six SNPs in the *PON* gene cluster explained 30.1% of variance in AREase activity (see Table 2). Addition of these SNPs, rs854567, rs2299257, rs2237583, rs2375005, rs3917486, and rs11768074 serially explained an additional 2.34%, 0.85%, 0.5%, 0.34%, 0.58%, and 0.26% of total variance in PON1 activity. Amongst these six SNPs, four SNPs were in *PON1,* one was in *PON2 *(rs2375005), and one was in *PON3 *(rs11768074); all are intronic.

Five of the six SNPs found to predict PON1 activity were the only SNPs in their bin (singletons). The sixth SNP, rs854567, was binned with one other typed SNP, rs2299260, *r* ^2^ = 0.80. To observe whether it was superior at predicting PON1 AREase activity, we replaced rs854567 with rs2299260 in the complete model of 10 SNPs plus covariates. The model including rs2299260 did not predict additional AREase activity as compared to the model including rs854567, with a total of 30.1% of AREase variance explained in the full model. Therefore, either SNP or an untyped SNP in LD may be the functional SNP resulting in the association identified.

To address the potential that untyped SNPs are the functional SNPs that underlie the identified AREase associations, the 1000 Genomes database for European ancestry was consulted via SNP-Nexus [68] for these six SNPs. Five of the six SNPs we found to predict PON1 AREase activity were not in strong LD (*r* ^2^ ≥ 0.8) with other regional SNPs, suggesting that they may be functional. Rs2375005, in contrast, is in strong LD with an additional five SNPs in *PON3* (*r* ^2^ = 0.901 with intronic rs978903 and synonymous A99A SNP rs1053275; *r* ^2^ = 0.837 with intronic rs10953146; *r* ^2^ = 0.81 for intronic rs11970910 and rs117154505) [10].

Prediction of POase activity utilizing these six SNPs that predicted AREase activity (including the base model with age, current smoking status, and the four functional *PON1* SNPs) resulted in 84.02% of POase enzymatic variance explained (see Table 3). This compared to 82.74% of variance explained with the base model with the four functional SNPs, age, and smoking status, with the high percentage of variation explained largely due to the effects of the *PON_Q192R_* polymorphism on paraoxon catalytic efficiency. Five of the six SNPs (excluding rs2237583) showed the same directionality of their effects as seen in the AREase analysis, and three had significant effects on POase: rs854567, rs2299257, and rs3917486. When creating a best-fit model that allowed any of the 51 SNPs studied to enter regression in addition to the base model, 84.96% of POase variance in activity was explained.

Similar application to the prediction of DZOase activity utilizing the six SNPs from the predictive AREase model plus the base model (age, current smoking status, and the four functional *PON1* SNPs) resulted in 54.85% of variance explained (see Table 4). Five of the six SNPs (excluding rs11768074) showed the same directionality of their effects, and 4 had significant effects (rs2299257, rs2237583, rs2375005, and rs3917486). When using the four functional SNPs, age, and sex alone, 50.99% of DZOase activity was explained. However, when allowing any of the 51 tagSNPs to enter the best-fit model, 55.60% of DZOase activity was accounted for, suggesting that different SNPs may affect DZOase.

We attempted to replicate SNPs previously identified by Carlson et al. as predicting PON1 activity in a nonoverlapping sample of 523 subjects (Table 5), because significance in an overlapping subset does not constitute replication. The SNPs identified by Carlson et al. were rs854549, rs3917564, rs2269829, rs854566, rs854572, and rs3917510. In our full analysis, rs854566 was tagged by rs854567 (*r* ^2^ = 0.93), which did enter the full model using the full sample and predicted 2.34% of AREase activity. Rs2269829 and rs854549 were not predictive of AREase in the full dataset. Rs3917564 was not included in the full model analysis due to minor allele frequency <0.01. Rs3917510 and rs854572 (*PON_−909_* promoter) were not tagged in the CVD chip analysis and were genotyped separately for the replication analysis. When we considered the independent sample to test the six Carlson SNPs in a linear model predicting AREase, which also included age, current smoking status, and the four functional *PON1* SNPs, two of the six Carlson et al. findings were replicated. Both rs854566 (Carlson *P* = 0.014, current rs854567 *P* = 1.64 × 10^−5^) and rs3917510 (Carlson *P* = 0.016, current *P* = 0.028) were significant in predicting AREase. Moreover, the direction of effect for rs854566 (Carlson coefficient = −10.6, current coefficient = −20.4) and rs3917510 (Carlson coefficient = 16.6, current coefficient = 14.3) were the same in both analyses.

None of the 10 SNPs identified in our full analyses, including the four previously known and the six newly reported to predict AREase, predicted CAAD status, considering the covariates censored age and current smoking status. Moreover, none of the SNPs had a *P* value <0.10. However, AREase activity, adjusted by age and current smoking status, was highly associated with CAAD status (*P* = 3.62 × 10^−6^), as previously reported in a smaller sample.

## 4. Discussion

Only four *PON1* SNPs are well established to affect PON1 activity. These mutations alone account for approximately only 25% of PON1 AREase activity, leaving a large amount of variation left unexplained. In this study, we utilized denser tagSNP genotyping and a 2.65-fold increased sample size than those previously used in the Carlson et al. study [27] to examine the effects of common variants, demonstrating the presence of additional functional genetic variance within the *PON* gene cluster. We identified six additional SNPs that predicted AREase activity (rs854567, rs2299257, rs2237583, rs2375005, rs3917486, and rs11768074). All are intronic, with four in *PON1* and one each in *PON2* (rs2375005) and *PON3* (rs11768074). Of these, only rs2375005 was found to be in strong LD with other regional SNPs in the 1000 genomes data, which included a *PON3* synonymous SNP (rs1053275). This LD block SNP is also reported to be in weaker LD with a *PON1_-1741GA_* promoter region polymorphism (maximum *r* ^2^ = 0.47) [34]. The remaining 5 SNPs associated with AREase may be functional or in weaker LD with a functional site. Rs854567 alone predicted 2.3% of the additional variance in AREase; it lies in the first intron of *PON1*, a common regulatory area.

For the many phenotypes with genetic associations to the PON cluster, knowledge of which SNPs are associated with functional changes is helpful in determining true associations from spurious ones. As discussed above, rs2375005 is in strong LD with an additional five SNPs in *PON3* (rs978903, rs1053275, rs10953146, rs11970910, and rs117154505). These include SNPs that have a reported association with sporadic amyotrophic lateral sclerosis [10]. In addition, Riedmaier et al. have demonstrated that a haplotype block including rs2375005 was associated with atorvastatin lactose hydrolysis and increased *PON1* mRNA expression in liver tissue [34]. Our results validate the presence of a functional SNP in this haplotype block.

In comparing these results to the six SNPs identified by Carlson et al, we replicate the effects of two SNPs, rs85466 and rs3917510, while failing to replicate four (Table 5) in nonoverlapping data. Rs854566 was represented in our analyses by the tagSNP, rs854567 (*r* ^2^ = 0.93). In contrast, the effects of rs854549, rs854572, rs3917564, and rs2269829 are not replicated here. Rs854572 is 5′ SNP *PON1_-909C/G_*; while it has been associated with AREase level, smaller studies suggested that all of its activity was attributable to LD with the four functional SNPs [66]. The Carlson et al. paper suggested that this site may have independent activity, but we find no additional effects of this site, in an independent sample of 523 subjects. In sum, our current study confirms both the effects of rs854566 or its bin-mate rs854567, predicting 2.3% of AREase activity and the effects of rs3917510, while also identifying five additional tagSNPs that accounted for approximately 2.7% of PON1 AREase activity that were not accounted for by Carlson et al.

The finding of *PON2* and *PON3* SNPs (rs2375005 and rs11768074, resp.) predicting PON1 AREase activity is intriguing. The *PON* genes are in a cluster and arranged in order from the centromere as *PON1, PON3*, and *PON2. *Each is transcribed in the same direction, toward the centromere. Therefore, variants in the* PON2* or *PON3* genes lie 5′ to *PON1*. Rs2375005 is in the sixth of eight *PON2* introns. Rs11768074 is in the last *PON3 *intron. Neither PON2 nor PON3 has intrinsic AREase activity [70], suggesting that these SNPs tag effects on PON1. As noted above, SNPs in the *PON3* rs2375005 haplotype block have been described to affect *PON1* mRNA level [34], thus the effects of these SNPs, or SNPs in LD with them, may regulate *PON1* expression.

Recent research in a cohort investigating SLE has linked rs17884563 and rs740264 in the *PON3* region [53] and five *PON2* SNPs [52] (rs6954345, rs13306702, rs987539, rs11982486, and rs4729189) with PON1 POase activity [52, 53]. These investigations utilized POase rather than AREase activity [71]; this is not optimal, as the *PON1_Q192R_* activity accounts for most POase activity. Of the *PON3* SNPs found to predict POase activity [53], both rs17884563 (intergenic between *PON2* and *PON3* in our annotation) and rs740264 were directly genotyped and included in our regression model for PON1 AREase activity but were not predictive. When applying rs17884563 or rs740264 to POase activity, which the aforementioned investigators used as their PON1 phenotype, neither was predictive of POase activity. For the* PON2* SNPs predictive of POase in the SLE cohort, all five were represented by tag SNPs (*r* ^2^ > 0.6), but only rs2375005 (*r* ^2^ = 1 with rs987539) was predictive of PON1 AREase activity. Interestingly, none of these five *PON2* SNPs predict POase in our data, including rs2375005 (*P* = 0.28). The differences in *PON2* and *PON3* SNP associations between our data and the SLE cohort may reflect differences in cohort selection criteria (older male vascular disease versus younger female SLE, cases and controls) or sizes (1,322 in our data versus 922 in the SLE data).

Application of the six SNPs from the AREase best-fit model to predicting POase and DZOase activity resulted in the prediction of 98.89% and 98.65% of enzymatic activity predicted by models, where all 51 SNPs were allowed to enter. Three of these six SNPs, all in *PON1*, also predict both PON1 POase and DZOase activities. While it is clear why coding SNPs would differentially influence the PON1 degradation of these three substrates, it is less clear why regulatory variants would. Further investigation is required to determine if and how these noncoding SNPs differentially influence PON1's multiple activities at the genomic, molecular, or cellular level.

None of the six new SNPs that predicted AREase activity were predictive of CAAD. In addition, none of the four functional *PON1* SNPs were predictive of CAAD, which is consistent with past findings with smaller sample sizes in this cohort [2, 3, 27]. Important sources of variance in AREase activity that are not captured by these genotypes or the covariates of age and current smoking must account for the strong association between this activity and CAAD. Possible sources of AREase variation include rare regional variants, regional gene regulation not captured by genotyping (such as methylation), variation in genes outside the *PON* cluster, nongenetic factors including statin drug use [72] and diet [73, 74], as recently reviewed [75], as well as interactions among these. Evidence of interactions includes the report of the association of *PON1* genotype and CHD only in subjects with diabetes [76]. These results emphasize the importance of researching the correlation of PON1 and cardiovascular disease more broadly by utilizing “PON status,” taking into account both the genotype of *PON1* SNPs and the plasma activity [11, 77], as well as investigating factors which affect the specific activity of PON1. PON1 has been suggested as a drug target for vascular and other diseases, thus a clear understanding of its role in disease is crucial [78].

Some limitations of this study must be considered. First, the study was comprised entirely of males of European descent, thereby limiting the generalizations that can be drawn from these findings. Second, this investigation considered only SNPs from the *PON* gene cluster. Variation in other genes may influence PON1 activity [79]. For example, peroxisome proliferator-activated receptor gamma (PPARG) activates PON1 expression in hepatocytes [80], leading to the possibility that variation in the *PPARG* gene could alter levels of PON1 protein. However, the larger size of this study and the denser tagSNPing of the *PON* cluster, relative to the earlier Carlson et al. work [27], allowed us to detect novel genetic variation that predicts *PON1* AREase activity.

In conclusion, our analysis of the *PON* gene cluster identifies six additional common genetic variants that predict AREase activity: four are novel, predicting 2.4% of AREase activity and two replicate past findings. The replicated SNPs include rs854567, which tags 2.3% of AREase variance, rs3917510, and rs2375005, which tags 0.3% of AREase variance. We do not identify additional effects of the *PON1_−909_* polymorphism. Future studies to quantify the role of rare genetic variation and variation outside the *PON* cluster on PON1 activity will be important. Finally, the continued lack of an association between *PON1, 2,* or* 3* genetic variants and CAAD, while PON1 activity is highly predictive, underscores the importance of utilizing PON status in future studies investigating the link between PON1 and vascular or other disease.

## Figures and Tables

**Table 1:** Characteristics of the 51 SNPs studied in the *PON* gene cluster.

SNP	Gene	Function^a^	Minor allele^b^	Major allele	MAF^c^
rs854549	*PON1*	3′-downstream	A	C	0.337
rs3735590	*PON1*	3′-UTR	A	G	0.060
rs3917577	*PON1*	3′-UTR	G	A	0.089
rs854552	*PON1*	3′-UTR	G	A	0.265
rs3917551	*PON1*	Intronic	A	G	0.051
rs3917550	*PON1*	Intronic	A	G	0.137
rs2269829	*PON1*	Intronic	G	A	0.278
rs3917542	*PON1*	Intronic	A	G	0.227
rs3917538	*PON1*	Intronic	A	G	0.236
rs2299257	*PON1*	Intronic	C	A	0.391
rs854560	*PON1*	Coding	T	A	0.360
rs3917498	*PON1*	Intronic	A	C	0.345
rs28699500	*PON1*	Intronic	G	A	0.289
rs854561	*PON1*	Intronic	A	G	0.357
rs854565	*PON1*	Intronic	A	G	0.294
rs2272365	*PON1*	Intronic	C	A	0.154
rs854567	*PON1*	Intronic	A	G	0.185
rs3917490	*PON1*	Intronic	A	G	0.490
rs2299261	*PON1*	Intronic	G	A	0.354
rs854568	*PON1*	Intronic	G	A	0.219
rs2299262	*PON1*	Intronic	A	G	0.399
rs854569	*PON1*	Intronic	A	C	0.216
rs2237583	*PON1*	Intronic	A	G	0.284
rs3917486	*PON1*	Intronic	A	G	0.054
rs3917481	*PON1*	Intronic	A	G	0.015
rs2237584	*PON1*	Intronic	A	G	0.058
rs3917478	*PON1*	Intronic	G	A	0.118
rs3917476	*PON1*	Intronic	A	C	0.031
rs854571	*PON1*	5′-upstream	A	G	0.289
rs13236941	*PON1*	5′-upstream	A	G	0.164
rs13228784	*PON1*	Intronic	G	A	0.255
rs17883513	*PON1*	Intronic	G	A	0.032
rs17886762	*PON1*	Intronic	A	G	0.072
rs17883952	*PON1*	Intronic	A	G	0.052
rs17884000	*PON3*	Intronic	G	A	0.202
rs9640632	*PON3*	3′-UTR	G	A	0.456
rs468	*PON3*	Intronic	G	A	0.066
rs11768074	*PON3*	Intronic	A	G	0.157
rs10487132	*PON3*	Intronic	G	A	0.390
rs740264	*PON3*	Intronic	C	A	0.254
rs17884563	*Intergenic*	*Intergenic*	T	A	0.109
rs17880030	*Intergenic*	*Intergenic*	A	G	0.199
rs17881071	*Intergenic*	*Intergenic*	A	G	0.198
rs2375005	*PON2*	Intronic	T	A	0.462
rs12026	*PON2*	Coding	C	G	0.240
rs2299264	*PON2*	Intronic	A	G	0.241
rs7803148	*PON2*	Intronic	A	G	0.405
rs2158806	*PON2*	Intronic	C	A	0.237
rs2286233	*PON2*	Intronic	A	T	0.131
rs10259688	*PON2*	Intronic	G	A	0.179
rs730365	*PON2*	Intronic	A	G	0.132

Abbreviations: UTR = untranslated region, MAF = minor allele frequency, intergenic = located between two gene regions.

^a^ SNP functional annotation from SNP-Nexus.

^b^ Major and minor allele annotation from the Illumina HumanCVD Bead Chip.

^c^ Minor allele frequencies calculated from the CLEAR study cohort.

**Table 2:** Best-fit model from stepwise linear regression predicting PON1 AREase activity.

Variable	Coefficient (± SE)	Gene^a^	MAF^b^	*t-*statistic^c^	AREase Variation %	*P*
(Intercept)	284.09 (±13.99)	—	—	20.304	—	< 2.0 × 10^−16^
*PON1_C-108T_*	−24.82 (±2.61)	*(PON1)*	0.43	−9.498	14.10%	< 2.0 × 10^−16^
*PON1_G-162A_*	4.61 (±4.60)	*(PON1)*	0.18	1.002	0.21%	0.317
*PON1_Q192R_*	−22.09 (±4.20)	*PON1*	0.33	−5.258	1.17%	1.8 × 10^−7^
*PON1_M55L_*	−7.05 (±3.64)	*PON1*	0.42	−1.94	1.01%	0.053
Age	−1.33 (±0.15)	—	—	−9.014	4.29%	< 2.0 × 10^−16^
Current smoker	−28.25 (±3.63)	—	—	−7.776	4.42%	1.95 × 10^−14^
rs854567	−8.19 (±4.77)	*(PON1)*	A = 0.185	−1.719	2.34%	0.086
rs2299257	12.66 (±3.57)	*(PON1)*	C = 0.391	3.546	0.85%	4.11 × 10^−4^
rs2237583	11.36 (±3.12)	(*PON1*)	A = 0.284	3.645	0.5%	2.82 × 10^−4^
rs2375005	−8.32 (±2.56)	*(PON2)*	T = 0.462	−3.25	0.34%	0.001
rs3917486	14.91 (±4.97)	(*PON1*)	A = 0.054	2.998	0.58%	0.003
rs11768074	8.42 (±4.48)	(*PON3*)	A = 0.157	1.878	0.26%	0.061

SE = standard error, MAF = minor allele frequency.

^a^Noncoding SNPs are presented in parentheses, for example, (*PON1).*

^b^Minor allele frequencies for the four functional SNPs reported through dbSNP. The remaining minor allele frequencies were calculated via the CLEAR cohort.

^c^ *t*-statistics and *P* values were calculated from the coefficients (from all subjects) and standard errors within the best-fit multivariate model by the glm function in R.

**Table 3:** Application of best-fit model for PON1 AREase activity to predict PON1 POase activity.

Variable	Coefficient (± SE)	Gene^a^	MAF^b^	*t-*Statistic^c^	POase Variation %	*P*
(Intercept)	29.36 (±1.17)	—	—	24.986	—	< 2.0 × 10^−16^
*PON1_C-108T_*	−1.91 (±0.22)	*(PON1)*	0.43	−8.762	11.78%	< 2.0 × 10^−16^
*PON1_G-162A_*	0.78 (±0.39)	*(PON1)*	0.18	2.023	3.93%	0.043
*PON1_Q192R_*	9.67 (±0.35)	*PON1*	0.33	27.27	65.61%	< 2.0 × 10^−16^
*PON1_M55L_*	−1.59 (±0.30)	*PON1*	0.42	−5.133	0.35%	3.5 × 10^−7^
Age	−0.09 (±0.01)	—	—	−7.475	0.78%	1.81 × 10^−13^
Current smoker	−1.27 (±0.31)	—	—	−4.155	0.31%	3.56 × 10^−5^
rs854567	−1.69 (±0.40)	*(PON1)*	A = 0.185	−4.246	0.54%	2.41 × 10^−5^
rs2299257	0.92 (±0.30)	*(PON1)*	C = 0.391	3.085	0.15%	0.002
rs2237583	−0.35 (±0.26)	(*PON1*)	A = 0.284	−1.347	0.09%	0.179
rs2375005	−0.23 (±0.21)	*(PON2)*	T = 0.462	−1.081	0.00%	0.28
rs3917486	2.20 (±0.42)	(*PON1*)	A = 0.054	5.271	0.47%	1.7 × 10^−7^
rs11768074	0.42 (±0.37)	(*PON3*)	A = 0.157	1.114	0.02%	0.266

SE = standard error, MAF = minor allele frequency.

^a^Non-coding SNPs are presented in parentheses, for example, (*PON1).*

^b^Minor allele frequencies for the four functional SNPs reported through dbSNP. The remaining minor allele frequencies were calculated via the CLEAR cohort.

^c^ *t*-statistics and *P* values were calculated from the coefficients (from all subjects) and standard errors within the best-fit multivariate model by the glm function in R.

**Table 4:** Application of best-fit model for PON1 AREase activity to predict PON1 DZOase activity.

Variable	Coefficient (± SE)	Gene^a^	MAF^b^	* t-*statistic^c^	DZOase Activity %	* P*
(Intercept)	154.26 (±4.24)	—	—	36.365	—	< 2.0 × 10^−16^
*PON1_C-108T_*	−8.69 (±0.79)	*(PON1)*	0.43	−11.054	12.82%	< 2.0 × 10^−16^
*PON1_G-162A_*	5.03 (±1.40)	*(PON1)*	0.18	3.597	5.10%	3.4 × 10^−4^
*PON1_Q192R_*	−20.41 (±1.28)	*PON1*	0.33	−15.944	23.71%	< 2.0 × 10^−16^
*PON1_M55L_*	−5.03 (±1.12)	*PON1*	0.42	−4.498	3.39%	7.75 × 10^−6^
Age	−0.44 (±0.44)	—	—	−9.797	4.21%	< 2.0 × 10^−16^
Current Smoker	−5.65 (±1.11)	—	—	−5.103	1.28%	4.08 × 10^−7^
rs854567	−2.26 (±1.44)	*(PON1)*	A = 0.185	−1.566	1.74%	0.118
rs2299257	4.03 (±1.08)	*(PON1)*	C = 0.391	3.73	0.70%	2.03 × 10^−4^
rs2237583	4.69 (±0.95)	(*PON1*)	A = 0.284	4.956	0.92%	8.57 × 10^−7^
rs2375005	−1.73 (±0.76)	*(PON2)*	T = 0.462	−2.258	0.22%	0.024
rs3917486	3.42 (±1.51)	(*PON1*)	A = 0.054	2.275	0.27%	0.023
rs11768074	−0.64 (±1.36)	(*PON3*)	A = 0.157	−0.466	0.01%	0.641

SE = standard error, MAF = minor allele frequency.

^a^Noncoding SNPs are presented in parentheses, for example, (*PON1).*

^b^Minor allele frequencies for the four functional SNPs reported through dbSNP. The remaining minor allele frequencies were calculated via the CLEAR cohort.

^c^ *t*-statistics and *P* values were calculated from the coefficients (from all subjects) and standard errors within the best-fit multivariate model by the glm function in R.

**Table 5:** Comparison of SNPs found significant in prior Carlson et al.^a^ study with current, non-overlapping sample.

SNP	Seattle SNP annotation	Carlson coefficient (±SE)	Carlson *t*-Statistic ^b^	Carlson *P ^c^*	Current coefficient (±SE)^d^	Current *t*-Statistic ^b^	Current *P * ^c^
rs854566^e^	*PON1_6842_*	−10.6 (±4.3)	−2.480	0.014	−20.4 (±4.68)	−4.353	1.64 × 10 − 5
rs3917510	*PON1_12471_*	16.6 (±6.9)	2.424	0.016	14.3 (±6.48)	2.208	0.028
rs2269829	*PON1_19470_*	−16.5 (±10.8)	−1.520	0.129	13.6 (±21.82)	0.625	0.533
rs3917564	*PON1_23887_*	−39.0 (±18.1)	−2.153	0.032	15.0 (±26.67)	0.564	0.573
rs854549	*PON1_29021_*	9.2 (±4.5)	2.051	0.041	−1.3 (±4.90)	−0.260	0.795
rs854572	*PON1_895_*	13.0 (±4.9)	2.677	0.008	−0.28 (±4.97)	−0.056	0.955

SE = standard error.

^a^Carlson et al. study *n* = 500 European male subjects [27].

^b^ *t*-Statistics and *P*-values were calculated from the coefficients from each subgroup (Carlson *n* = 500, current study *n* = 523) and standard errors within the best-fit multivariate model by the glm function in R.

^c^Both Carlson and current study utilized a linear regression model adjusting for age, current smoking status, and the four functional PON1 SNPs.

^d^Current study subset of 523 European male subjects not considered by Carlson et al.

^e^Represented by proxy SNP, rs854457, with LD *r* ^2^ = 0.93 in the current study.

## Abbreviations

AIC: Akaike's Information Criterion
CAAD: Carotid artery disease
CHD: Coronary heart disease
CLEAR: Carotid Lesion Epidemiology and Risk cohort
DZOase: Diazoxon enzymatic hydrolysis
POase: Paraoxon enzymatic hydrolysis
PON: Paraoxonase
tagSNP: Tagging single-nucleotide polymorphism.
